# STARTing to dissect the molecular determinants of GLABRA2 activity

**DOI:** 10.1093/plphys/kiac436

**Published:** 2022-09-22

**Authors:** Sergio Galindo-Trigo

**Affiliations:** Section for Genetics and Evolutionary Biology, Department of Biosciences, University of Oslo, 0316 Oslo, Norway

The epidermis in plants is the outermost cell layer and as such is crucial to mediate the plant’s interaction with its environment. In aerial tissues like leaves and stems, the epidermis protects the plant from the threats of pathogens, herbivores, and unfavorable environmental conditions. Meanwhile, the root epidermis actively increases the roots’ surface to favor water and nutrient intake, as well as to establish symbiotic relationships with beneficial fungi.

To carry out such a diverse number of functions, epidermal cells can differentiate into aerial hairs or trichomes, stomata, or root hairs, among others. In some plants, trichomes act as a direct defence mechanism as they accumulate herbivore-deterrent chemicals, while in Arabidopsis (*Arabidopsis thaliana*) trichomes act as mechanosensors, initiating Ca^2+^ waves that reach and alert distant cells of the presence of a threat (Matsumura et al., 2022). However, not all epidermal cells differentiate into trichomes or root hairs. Specific genes regulate epidermal cell fate and therefore the ratio of epidermal cell types (Zuch et al., 2022). For example, *GLABRA2* (*GL2*) encodes a transcription factor (TF) and is one of the best characterized regulators of epidermal cell fate in Arabidopsis, controlling trichome differentiation, root hair-less cell file determination, as well as mucilage synthesis in seeds (for review, see Ariel et al., 2007).

TFs bind specific DNA sequences (or target sites) and execute changes in the expression of genes, usually by recruiting additional TFs and components of the basal transcriptional machinery. TFs are modular molecules comprising distinct protein domains, each of which typically contributes to the TF activity in a different manner: the DNA binding domain determines the DNA sequence to which the TF will bind, whereas other protein domains may influence the TF’s protein interactome, intracellular transport dynamics, and protein stability. Distinguishing the contributions of individual protein domains in a TF is crucial to harness their full potential to fine-tune transcription for better yields or to efficiently design synthetic transcriptional networks (Khalil et al., 2012).

GL2 belongs to the homeodomain-leucine zipper (HD-ZIP) class IV TF family that has a plant-specific domain arrangement consisting of an HD-ZIP DNA-binding domain tandem near the N-terminus, followed by a steroidogenic acute regulatory protein-related lipid-transfer (START) domain, and a START-associated domain (STAD) near the protein C-terminus (Schena and Davis, 1992). In animals and the liverwort *Marchantia polymorpha*, the START domain binds to lipids and influences their transport (Clark, 2020; Hirashima et al., 2021). Nevertheless, the functional contribution of the START domain to GL2 transcriptional activity has remained speculative until now.

In the current issue of *Plant Physiology*, Mukherjee *et al.* (2022) molecularly dissect and characterize the roles of the START domain in GL2 function as well as its interactions with the better-characterized HD-ZIP domains. The approach taken by Mukherjee *et al.* (2022) involved designing a comprehensive set of *GL2* mutant variants that could potentially impair the HD and START domain activities. Knockout *gl2* plants were transformed with the set of *GL2* variants fused to a fluorescent protein, and their individual capacity to rescue the well-characterized *gl2* developmental defects was scored. Confirming the predictive power of homology-based structural models, all mutant variants of *GL2* failed to rescue *gl2* trichome development, root hair density, and seed mucilage accumulation, indicating that the START domain, similarly to HD, is indispensable for GL2 transcriptional function. Similar phenotypic defects were quantified in domain-specific START and HD mutants, indicating that both domains are equally necessary for GL2 activity.

HDs allow DNA binding and dictate the target site of TFs. Consequently, and based on genetic complementation assays, the START domain must govern another equally indispensable molecular mechanism for GL2 activity. The authors first analyzed the subcellular localization of the mutant GL2 variants and concluded that neither the HD nor START domains impair GL2 nuclear localization. Next, the importance of the START domain for the ability of HD-ZIP(IV) proteins to bind DNA was tested in electrophoretic mobility shift assays (EMSAs). Due to difficulties expressing recombinant GL2, a close relative, PROTODERMAL FACTOR2 (PDF2), and PDF2 mutant variants equivalent to those designed for GL2 were used in these experiments. EMSAs and western blots indicated that the START domain of PDF2 does not influence the TF DNA binding in vitro and that the START domain is necessary for homodimerization.

A ChIP-seq experiment using several GL2 mutant variants revealed that the presence or functionality of the START domain does not substantially alter GL2 DNA binding specificity in vivo. Yeast-two hybrid and split-fluorescent protein assays using a comprehensive set of full-length and domain deletion GL2 variants confirmed the requirement of the START domain for effective GL2 homodimerization in yeast and in planta.

Finally, Mukherjee and colleagues investigated whether the START domain could influence protein stability by applying protein synthesis inhibitors to seedlings expressing different GL2 mutant variants and quantifying the corresponding GL2 protein in a time-course experiment. The latter experiment clearly indicated that GL2 variants lacking a functional START domain were degraded more rapidly than wild-type GL2. Using a proteasome degradation inhibitor extended the lifetime of START-domain mutant GL2 variants several hours, suggesting that the proteasome degradation pathway could be controlling GL2 protein stability.

The authors therefore concluded the START domain is vital for HD-ZIP(IV) TF activity as it (i) enables homodimerization, therefore likely allowing a more effective recruitment of the basal transcriptional machinery; and (ii) increases protein stability, increasing the active window for each HD-ZIP(IV) molecule to conduct its cellular activity before protein turnover. Moving forward it would be very informative to address the lipid-binding capacity of the HD-ZIP(IV) START domains by means of biochemical and evo-devo approaches, that is, testing their capacity to directly bind collections of lipids, and investigating whether they can genetically complement the previously identified START domain of the *M. polymorpha* STAR2 protein. Pinpointing the type of lipid and the conditions under which it is bound by HD-ZIP(IV) TFs would provide us with a clearer picture of how these TFs are mechanistically regulated and perhaps reveal how these TFs may perceive and respond to readouts of cellular metabolism and/or important small signaling lipids.

*Conflict of interest statement*. None declared.
